# Harboring CAR-T cells using an injectable scaffold to treat solid tumors

**DOI:** 10.1093/nsr/nwae075

**Published:** 2024-02-28

**Authors:** Li Tang

**Affiliations:** Institute of Bioengineering (IBI), École polytechnique fédérale de Lausanne (EPFL), Switzerland

Genetic expression of chimeric antigen receptors (CAR) enables T cells to target and kill cancer cells in an MHC-independent manner, resulting in a potent antitumor response [1]. To date, more than 10 CAR-T cell products have been approved globally for the treatment of hematologic disorders such as relapsed/refractory B-cell acute lymphoblastic leukemia, non-Hodgkin lymphoma, and multiple myeloma [2,3]. Unfortunately, the clinical efficacy of CAR-T cells in solid tumors, which encompasse over 90% of the entire tumor classification, is limited [4,5]. Recently, engineering approaches such as cell reservoir or microneedle patch for delivery of CAR-T cells *in vivo* have shown some promise in improving the efficacy against solid tumors [6,7]. However, a biomimetic cell delivery strategy using an injectable scaffold for enhanced proliferation and function of CAR-T cells has not been attempted.

**Figure 1.**
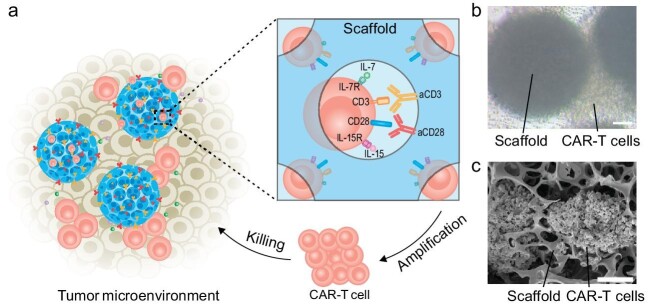
The illustration depicts the enhancement of CAR-T cell therapy against solid tumors using a lymph node-biomimetic injectable scaffold. (a) A schematic representation of the activation and expansion of CAR-T cells within the scaffold. (b) Representative optical microscope images and (c) scanning electron microscope images demonstrate the successful activation and expansion of CAR-T cells within the porous scaffold. Scale bar, 50 μm. Adapted from Ref. [8].

Recently, a study published in *National Science Review* by Zhen Gu and colleagues proposed a lymph node-biomimetic injectable scaffold designed for *in situ* activation and expansion of CAR-T cells within cervical carcinoma, thereby improving the persistence and effectiveness of CAR-T cells for the treatment of solid tumors (Fig. 1a) [8]. The scaffold comprises a three-dimensional porous structure incorporating potent T-cell activation signals. Utilizing microfluidic technology, porous poly(lactic-co-glycolic acid) (PLGA) microspheres were fabricated and equipped with anti-CD3 antibodies, anti-CD28 antibodies, interleukin (IL)-7, and IL-15. Similar to the reticular microarchitecture of lymph nodes that provides venues for T cell activation and proliferation, the biomimetic scaffold facilitates the stimulation and preservation of CAR-T cells (Fig. 1b and c). Consequently, the scaffold led to a 50-fold expansion of CAR-T cells *in vitro* and a 15-fold expansion *in vivo*. Importantly, CAR-T cells activated by the scaffold exhibited enhanced anti-tumor activity compared to those activated by traditional methods. Additionally, the microscale scaffold can be directly injected into tumors, eliminating the need for additional surgical procedures and enhancing patient comfort during clinical translation.

In conclusion, the insightful research led by Gu's team has introduced a novel approach to augment the infiltration, expansion, and anti-tumor efficacy of CAR-T cells, highlighting the potential of biomaterial-enabled CAR-T therapy in treating solid tumors. Although the current study revolves around CAR-T cell delivery, this biomimetic system is anticipated to serve as a versatile platform for delivering a wide range of cell-based therapies signifying broad prospects for applications.
